# Managing Kartagener's Syndrome With Type II Respiratory Failure and Left-Sided Pneumothorax During the COVID-19 Pandemic: A Case Report

**DOI:** 10.7759/cureus.44632

**Published:** 2023-09-04

**Authors:** Muhammad Leel, Marvi Abid, Kiran Fatima, FNU Sandesh, Aakash Kumar

**Affiliations:** 1 Infectious Diseases, District Headquarter Hospital, Bhakkar, PAK; 2 Internal Medicine, Jinnah Postgraduate Medical Centre, Karachi, PAK; 3 Internal Medicine, Jinnah Sindh Medical University, Karachi, PAK; 4 Internal Medicine, Mercy Health — St. Rita's Medical Center, Lima, USA

**Keywords:** kartagener's syndrome, situs inversus, respiratory insufficiency, pneumothorax, covid-19 pandemic, ciliary motility disorders

## Abstract

Kartagener's syndrome is an autosomal recessive disorder with symptoms varying from chronic sinusitis to bronchiectasis and situs inversus (a congenital condition in which the visceral organs are located in an opposite location). We describe a rare and complicated case of a 40-year-old female patient who presented to the emergency room with significant chest congestion and Kartagener's syndrome. This case demonstrates the value of individualized and proactive care as well as the challenge of managing this illness, particularly when it coexists with type II respiratory failure related to pneumothorax.

## Introduction

Kartagener’s syndrome is an inherited autosomal recessive disorder, classified as a subtype of primary ciliary dyskinesia (PCD), resulting in malfunction in the cilia in lungs and other visceral organs [1,2]. This is a rare disease with a prevalence ranging from 1/15000-20000 to 1/32000 [2,3]. Interestingly, the prevalence is estimated to be higher in communities with common consanguineous marriages [1-4]. It presents as a combination of situs inversus (a condition where major body organs are mirrored to their original placement), bronchiectasis, and sinusitis [4].

Patients often suffer from repeated bouts of respiratory infections [5]. This disorder is a result of multiple mutations in various genes, including DNAH5 and DNAI1, which are pivotal in ciliary motion [6]. Moreover, these genes are not the only ones responsible for this disorder, as evidence has identified that some other genetic factors might also contribute to the severity of this ciliary dysfunction [7,8]. Symptoms such as respiratory distress and repeated lung infections are prominent findings since birth in these patients; however, severity may vary across individuals.

The diagnosis of Kartagener’s syndrome is made on the basis of both clinical and radiological findings. Dextrocardia, which is considered a hallmark sign of Kartagener’s syndrome, can be detected by chest radiography. Computed tomography (CT) of the face and chest can detect mucosal thickening in the sinuses and the characteristic "tree-in-bud" bronchiectasis, which are consistent with this condition. Situs inversus is confirmed on a CT scan, where the liver is on the left and the spleen is on the right. In such cases, laboratory testing does not reveal significant information; however, sometimes microorganisms are detected in sputum cultures [9,10].

Complications associated with Kartagener's syndrome, including pneumothorax, are uncommon yet can be life-threatening. Some cases have documented multiple pulmonary issues such as pulmonary tuberculosis and aspergilloma [11,12]. The prognosis of Kartagener's syndrome has been notably enhanced through a combination of treatment strategies, encompassing antibiotics, physiotherapy, and prompt surgical interventions [9-12].

## Case presentation

A 40-year-old female with a known case of Kartagener’s syndrome presented to the emergency department of a tertiary care hospital with complaints of shortness of breath, chest congestion, vomiting, and cough persisting for five days. Upon arrival, her oxygen saturation was at 82% on five liters of supplemental oxygen, and she exhibited a state of mild dyspnea and lethargy.

An immediate admission to the pulmonology ward was facilitated on 23 January 2021. Prior to the admission to the pulmonology ward, SARS-Cov-2 RT-PCR was done, which came back negative. Initial physical assessment revealed bilateral crepitations with decreased lung sounds on the left side, and a Glasgow coma score of 12/15 suggestive of a mild to moderate neurological deficit. A thorough comprehensive systematic examination was done which yielded no other significant findings.

Imaging and lab investigations, including chest radiographs and high-resolution computed tomography (HRCT), revealed dextrocardia, bilateral infiltrates, and a left-sided pneumothorax which were in line with her Kartagener’s syndrome. These also showed signs of hyperinflation in both lungs, fibrocalcific scarring, basilar atelectasis, and mild to moderate pleural effusion. Bronchiectasis was prominently found in the left lung. The possibility of allergic bronchopulmonary aspergillosis could not be excluded from HRCT. Arterial blood gases were indicative of respiratory acidosis.

**Figure 1:**
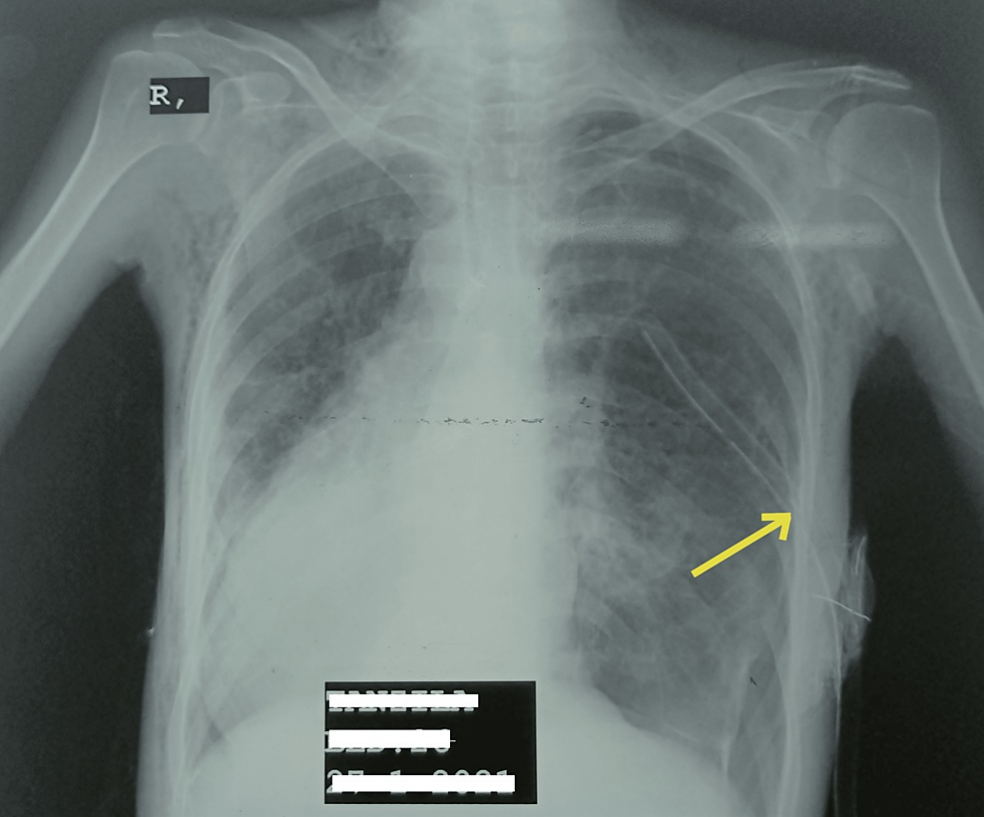
Chest radiograph revealing successful chest tube insertion

Sputum samples were sent for bacterial and fungal infections. The sputum culture indicated a resistant strain of Staphylococcus aureus, while an AFB smear and GeneXpert did not detect any Mycobacterium tuberculosis. Informed consent was obtained for chest tube placement in the pleural cavity, which was successfully carried out without complications as observed in X-ray (Figure 1).

Post-procedure management included pain management and administration of broad-spectrum antibiotics, proton-pump inhibitors, and corticosteroid therapy. To address the respiratory acidosis, the patient was also prescribed bilevel positive airway pressure (BIPAP) therapy with concomitant physiotherapy.

By 27 January 2021, the patient showed signs of stability, maintaining 100% oxygen saturation with BIPAP. However, she continued to produce significant amounts of sputum. A subsequent sputum culture sensitivity test on 31st January did not reveal any growth of microorganisms.

After three days, the patient's condition had further stabilized, and she opted for leave against medical advice (LAMA). Before discharge, she was thoroughly counseled about the importance of regular physiotherapy and the necessity of consistent follow-up appointments to ensure continued management of her condition.

## Discussion

Our case describes a patient with Kartagener’s syndrome, presenting with left-sided pneumothorax and mild pleural effusion leading to a type II respiratory failure. The patient was immediately placed on supplemental oxygen, followed by chest tube placement and pain management. The patient was started on antibiotics after which sputum cultures yielded positive Staphylococcus aureus. Additionally, to maintain adequate oxygenation, BIPAP therapy was initiated. The patient was started on corticosteroids and proton-pump inhibitors as supportive measures to improve prognosis.

Patients suffering from Kartagener’s syndrome suffer from recurrent pulmonary infections, for which medical attention is sought. On a pathophysiological level, the condition is associated with the accumulation of secretions which lead to bronchiectasis, sinusitis, and airflow obstruction resulting in respiratory failure [13], which was seen in our case.

No definitive treatment exists for Kartagener’s syndrome, a genetically acquired disease. Instead, managing these patients is symptomatic and generally constitutes antibiotics to treat recurrent respiratory infections. Ibrahim and Daood reported a Kartagener’s syndrome case involving a three-year-old boy who had presented to the Emergency Department with respiratory failure. As was the case with our patient, the patient reported by Ibrahim and Daood could also not maintain oxygen saturation. The initial approach to the patient in the case was the use of bronchodilators and corticosteroids. Due to a lack of response to this treatment regimen, the patient was oxygenated using a facial mask with a partial rebreathing reservoir, which also failed to maintain adequate oxygenation. A similar approach to management was then employed, where the patient was continued on corticosteroids, antibiotics, and non-invasive ventilation. However, upon failure to improve respiratory acidosis, the patient had to be intubated [14].

Pneumothorax is an expected but rather rare feature in patients suffering from Kartagener’s syndrome. A similar case of Kartagener’s syndrome was also reported by Prabhakar et al., where the patient presented with left-sided pneumothorax, as was the feature observed in our case. However, the patient followed by Prabhakar et al. reported a normal cognitive function with a normal Glasgow coma scale with no significant neurological deficit. The patient was managed by inserting a left-sided drain attached to an underwater seal. Additionally, a combined drug therapy using corticosteroids and long-acting beta-two agonists combined with N-acetylcysteine improved his condition dramatically [15].

However, in another case of Kartagener’s syndrome reported by Wang et al., a similar combined therapy using antibiotics, oral steroids, and bronchodilators was administered, which did not bring about desirable results [16]. This failure in drug response could be associated with end-stage bronchiectasis, which was observed in the patient. In this case, a double lung transplantation was performed, bringing positive results throughout the follow-up. 

Kartagener’s syndrome is often overlooked as a potential cause in patients with upper or lower respiratory tract infections. In our case, the first approach after observing symptoms of possible respiratory failure was to evaluate the patient for COVID-19, which was more prevalent at the time. However, in this patient, the type II respiratory failure was secondary to left-sided pneumothorax. This case highlights the challenges associated with the management of patients who are suffering from complications of Kartagener’s syndrome.

## Conclusions

This case highlights the importance of an individualized and prompt management strategy that is necessary due to the frequency of complications patients with Kartagener’s syndrome suffer from. The effective treatment of this patient highlights the need for thorough diagnosis, quick intervention, and customized therapy approaches. The knowledge gained from this case adds to our overall understanding of Kartagener's syndrome and can help guide future treatment procedures for patients with comparable symptoms.
